# Short- and long-term dynamics of gut microbiota, highlighting *Fusobacterium nucleatum*, *Parvimonas micra*, and *Peptostreptococcus stomatis* after colorectal cancer resection: prospective cohort study

**DOI:** 10.1093/bjsopen/zrag037

**Published:** 2026-05-21

**Authors:** Tomosuke Mukoyama, Kimihiro Yamashita, Masafumi Saito, Mitsugu Fujita, Seiichi Omura, Takuo Emoto, Tomoya Yamashita, Masayuki Ando, Kyosuke Agawa, Kota Yamada, Akihiro Watanabe, Tomoki Abe, Takao Tsuneki, Yukari Adachi, Ryuichiro Sawada, Yasufumi Koterazawa, Hitoshi Harada, Naoki Urakawa, Hironobu Goto, Hiroshi Hasegawa, Shingo Kanaji, Takeru Matsuda, Taro Oshikiri, Yoshihiro Kakeji

**Affiliations:** Division of Gastrointestinal Surgery, Department of Surgery, Graduate School of Medicine, Kobe University, Kobe, Hyogo, Japan; Division of Gastrointestinal Surgery, Department of Surgery, Graduate School of Medicine, Kobe University, Kobe, Hyogo, Japan; Department of Biophysics, Kobe University Graduate School of Health Sciences, Kobe, Japan; Department of Immunology and Microbiology, National Defence Medical College, Tokorozawa, Japan; Center for Medical Education and Clinical Training, Kindai University Faculty of Medicine, Osaka-Sayama, Osaka, Japan; Department of Microbiology, Faculty of Medicine, Kindai University, Osaka-Sayama, Osaka, Japan; Division of Cardiovascular Medicine, Department of Internal Medicine, Kobe University Graduate School of Medicine, Kobe, Japan; Division of Cardiovascular Medicine, Department of Internal Medicine, Kobe University Graduate School of Medicine, Kobe, Japan; Division of Advanced Medical Science, Technology and Innovation, Kobe University Graduate School of Science, Kobe, Japan; Division of Gastrointestinal Surgery, Department of Surgery, Graduate School of Medicine, Kobe University, Kobe, Hyogo, Japan; Division of Gastrointestinal Surgery, Department of Surgery, Graduate School of Medicine, Kobe University, Kobe, Hyogo, Japan; Division of Gastrointestinal Surgery, Department of Surgery, Graduate School of Medicine, Kobe University, Kobe, Hyogo, Japan; Division of Gastrointestinal Surgery, Department of Surgery, Graduate School of Medicine, Kobe University, Kobe, Hyogo, Japan; Division of Gastrointestinal Surgery, Department of Surgery, Graduate School of Medicine, Kobe University, Kobe, Hyogo, Japan; Division of Gastrointestinal Surgery, Department of Surgery, Graduate School of Medicine, Kobe University, Kobe, Hyogo, Japan; Division of Gastrointestinal Surgery, Department of Surgery, Graduate School of Medicine, Kobe University, Kobe, Hyogo, Japan; Division of Gastrointestinal Surgery, Department of Surgery, Graduate School of Medicine, Kobe University, Kobe, Hyogo, Japan; Division of Gastrointestinal Surgery, Department of Surgery, Graduate School of Medicine, Kobe University, Kobe, Hyogo, Japan; Division of Gastrointestinal Surgery, Department of Surgery, Graduate School of Medicine, Kobe University, Kobe, Hyogo, Japan; Division of Gastrointestinal Surgery, Department of Surgery, Graduate School of Medicine, Kobe University, Kobe, Hyogo, Japan; Division of Gastrointestinal Surgery, Department of Surgery, Graduate School of Medicine, Kobe University, Kobe, Hyogo, Japan; Division of Gastrointestinal Surgery, Department of Surgery, Graduate School of Medicine, Kobe University, Kobe, Hyogo, Japan; Division of Gastrointestinal Surgery, Department of Surgery, Graduate School of Medicine, Kobe University, Kobe, Hyogo, Japan; Division of Gastrointestinal Surgery, Department of Surgery, Graduate School of Medicine, Kobe University, Kobe, Hyogo, Japan; Division of Gastrointestinal Surgery, Department of Surgery, Graduate School of Medicine, Kobe University, Kobe, Hyogo, Japan; Division of Gastrointestinal Surgery, Department of Surgery, Graduate School of Medicine, Kobe University, Kobe, Hyogo, Japan

## Abstract

**Background:**

Colorectal cancer is associated with gut microbiota alterations, including enrichment of colorectal cancer-associated bacteria such as *Fusobacterium nucleatum*, *Parvimonas micra*, and *Peptostreptococcus stomatis*. However, the impact of curative resection on their postoperative dynamics remains unclear.

**Methods:**

Faecal samples were collected from patients with colorectal cancer and controls. Samples were collected before surgery and at 1 week, 1 month, and 6 months after surgery. Microbial diversity was evaluated using 16S ribosomal RNA sequencing, and *F. nucleatum*, *P. micra*, and *P. stomatis* were quantified by quantitative polymerase chain reaction.

**Results:**

A total of 83 faecal samples were collected from 28 patients with colorectal cancer and 16 healthy controls. Alpha diversity decreased at 1 week and recovered by 1 month. Unweighted unique fraction metric suggested recovery of community membership by 6 months, whereas Bray–Curtis and weighted unique fraction metric remained altered, indicating persistent community reorganization. At the class level, Negativicutes and Clostridia decreased at 1 week and gradually recovered, whereas Bacilli showed a transient increase. In contrast, *F. nucleatum*, *P. micra*, and *P. stomatis* declined significantly after surgery and remained suppressed at 6 months. Patients who developed recurrence had higher preoperative *P. micra* and *P. stomatis* levels, though differences were not observed after surgery.

**Conclusion:**

Colorectal cancer resection induces an acute postoperative disruption of the gut microbiota, followed by recovery of overall diversity and major taxonomic features, but stabilization in a state that does not fully recapitulate the preoperative microbiota. Colorectal cancer-associated bacteria (*F. nucleatum*, *P. micra*, and *P. stomatis*) remained persistently reduced after surgery, highlighting their dependence on the tumour microenvironment. Monitoring these bacteria in faecal samples may offer a non-invasive means to assess microbiota recovery and potential recurrence risk.

## Teaser

Curative colorectal cancer resection caused rapid perioperative gut microbiota changes, including transient reductions in diversity. Colorectal cancer-associated bacteria (*Fusobacterium nucleatum*, *Parvimonas micra*, and *Peptostreptococcus stomatis*) were markedly decreased after surgery and remained suppressed for at least 6 months. These findings suggest that tumour-associated bacteria depend on the tumour microenvironment and may serve as stool-based biomarkers for monitoring recovery and recurrence risk.

## Introduction

Colorectal cancer (CRC) is the third most common cancer worldwide and a leading cause of cancer-related death. Its incidence is expected to continue rising in the coming decades^1^. Increasing evidence shows that gut microbiota alterations contribute to colorectal tumorigenesis through mechanisms such as chronic inflammation, immune modulation, and tumour-promoting signalling pathways^2^.

Several CRC-associated microbial signatures have been identified. Among them, *Fusobacterium nucleatum* and enterotoxigenic *Bacteroides fragilis* are frequently enriched in faecal and mucosal samples from patients with CRC^3–5^. These bacteria promote tumorigenesis in experimental models and are therefore considered CRC-associated^6,7^. High intratumoral *F. nucleatum* after neoadjuvant therapy has also been linked to recurrence and poor prognosis^8,9^. In addition, oral bacteria such as *Parvimonas micra* and *Peptostreptococcus stomatis* are often increased in patients with CRC, further supporting their role in tumour development^10^.

Despite these associations, little is known about how the gut microbiota changes after curative CRC resection. Previous studies have reported shifts in taxa such as *Fusobacterium*, Ruminococcaceae, and Clostridiales^11,12^, but findings have been inconsistent due to differences in design, follow-up, and bacterial targets. Perioperative factors such as mechanical bowel preparation and antibiotic use are also known to disrupt the gut microbiota^13–15^. These effects are usually considered transient, with microbial composition typically recovering to baseline within weeks to 1 month^16,17^. However, the exact timeline for full restoration and the specific bacterial taxa most affected remain unclear^14,16,17^, particularly CRC-associated bacteria such as *F. nucleatum , P. micra*, and *P. stomatis*.

To address this gap, a prospective study was designed to characterize short- and long-term changes in the gut microbiota of patients undergoing curative CRC resection. By tracking overall community diversity, as well as the dynamics of *F. nucleatum*, *P. micra*, and *P. stomatis*, the aim was to determine whether these species are persistent after resection and whether their suppression reflects dependence on the tumour microenvironment.

## Methods

### Sample collection

This study was approved by the Research Ethics Committee of Kobe University Hospital (No. B210121), and written informed consent was obtained from all participants. Faecal samples were collected from patients with pathologically confirmed CRC undergoing curative laparoscopic resection, and from age- and sex-matched healthy controls. Exclusion criteria included recent antibiotic use (within 1 month before sampling), distant metastases, multiple cancers, inflammatory disease, systemic autoimmune disorders, or infections.

Samples were obtained before surgery, and at 1 week, 1 month, and 6 months after surgery. Patients underwent mechanical bowel preparation before surgery with magnesium citrate and received a perioperative β-lactam antibiotic (cefmetazole) until postoperative day 1. All samples were collected in Metabolokeeper® solution (TechnoSuruga Lab, Japan), stored at room temperature, and transported to TechnoSuruga Lab Co., Ltd. for DNA extraction.

### DNA extraction, 16S ribosomal (r)RNA gene sequencing, and bioinformatic processing

DNA was extracted using an automated DNA isolation system (GENE PREP STAR PI-480®, KURABO, Osaka, Japan), as described previously^18^. The V3–V4 regions of bacterial and archaeal 16S rRNA were amplified with Pro341F/Pro805R primers and a dual-index method^18,19^. Amplicons were sequenced on a MiSeq system (2 × 301 base pairs (bp) with MiSeq Reagent Kit v3 (600-cycle). Primer sequences were trimmed using Cutadapt (v1.18)^20^. Primer-trimmed paired-end reads were then quality filtered, denoised, merged, and chimera-filtered using the DADA2 plugin implemented in QIIME 2 (v2022.2)^21^. Based on inspection of per-base quality score profiles, forward and reverse reads were truncated at 250 bp and 230 bp, respectively (*Fig. S1*). An expected error threshold of five was applied to balance sequence quality and read retention. Chimeric sequences were removed using the consensus method. This procedure resulted in high-resolution amplicon sequence variants (ASVs). Representative sequences were then generated. Taxonomy was assigned using the Greengenes2 Database (2022.10) with a Naive Bayes classifier^22^.

### Diversity analyses

Alpha diversity was assessed at the ASV level using the Shannon diversity index and Chao1 richness. Longitudinal changes within the CRC cohort were analysed using linear mixed-effects models (LMMs), as described in the Statistical analysis section. Healthy controls, which were sampled only once, were included as an independent reference group for descriptive comparison but were not incorporated into the longitudinal models. For beta diversity analyses, ASV-level feature tables were collapsed to the genus level before distance calculation. Bray–Curtis, weighted unique fraction metric (UniFrac), and unweighted UniFrac distances were calculated and compared statistically in R (v4.5.2). The following packages were used for downstream analyses: qiime2R (v0.99.6), phyloseq (v1.38.0), and MicrobeR (v0.3.2). UniFrac distances were calculated in R using the phyloseq package based on the phylogenetic tree inferred in QIIME 2 and imported via qiime2R. Pairwise PERMANOVA was performed using the adonis2 function in the vegan package (v2.6.4). For each group comparison, analyses were restricted to samples from the two groups being compared. To account for repeated measures within individuals, permutations were constrained within subjects using patient identification (ID) as a strata variable. Resulting *P* values were adjusted for multiple testing using the Benjamini–Hochberg false discovery rate procedure. Figures were generated using the ggplot2 package (version 3.4.2).

### Longitudinal analysis of postoperative microbiota dynamics

To summarize postoperative temporal restructuring at higher taxonomic ranks, time-associated changes in relative abundance were assessed at the phylum and class levels. ASV counts were agglomerated to the respective taxonomic levels and converted to relative abundances. Values were log-transformed after addition of a small pseudocount (log [RA + 1 × 10^−6^]) before analysis. Longitudinal changes were evaluated using LMMs as described in the Statistical analysis section. Model-derived estimated marginal means were used to quantify changes relative to the preoperative baseline across postoperative time points. Taxa were ranked according to the maximum absolute change observed and selected for visualization to highlight the most dynamic features. Genus-level analyses were restricted to the class exhibiting the largest postoperative shifts. Within the selected class, genera agglomerated from ASV counts were ranked according to the magnitude of estimated directional changes between adjacent time points. Specifically, absolute changes between the preoperative–1 week and 1 week–6 month groups were calculated for each genus, and the smaller of the two values was used as a ranking metric to prioritize genera exhibiting consistent perioperative dynamics rather than transient fluctuations. Genera showing the largest changes were summarized descriptively. In addition to the longitudinal modelling described above, genus-level cross-sectional differences among groups were explored using linear discriminant analysis effect size (LEfSe)^23^. LEfSe was applied to compare relative abundances of genera among the healthy control, preoperative, and 6 month groups. Analyses were performed using the official LEfSe Docker image following the developer’s instructions (https://huttenhower.sph.harvard.edu/lefse/). LEfSe employs non-parametric statistical tests (Kruskal–Wallis and Wilcoxon rank-sum tests) to identify features with differential relative abundance among groups, followed by linear discriminant analysis (LDA) to estimate the effect size of each feature. Genera with an LDA score > 2.0 and a nominal *P* < 0.050 were considered enriched and are reported as exploratory findings, complementary to the primary longitudinal analyses^24^.

### Quantitative polymerase chain reaction (qPCR)

qPCR was performed on residual genomic DNA to quantify CRC-associated bacteria (*Table 1*)^10,24,25^. Universal primers targeted the *tuf* gene, which encodes elongation factor Tu (EF-Tu). As EF-Tu is highly conserved and usually present in one or two copies per chromosome, *tuf* served as a reliable reference^26^. Reactions were run in a Thermal Cycler Dice Real-Time PCR System TP860® (Takara Bio Inc., Shiga, Japan). Amplification for *tuf* detection was performed in duplicate in 25 μl volumes containing 12.5 μl of 2× TB Green Premix Ex Taq GC, 5 μl of 5× *tuf* Primer Mix, 5.5 μl of water, and 2 μl of genomic DNA (20 ng). Reactions for *F. nucleatum*, *P. micra*, and *P. stomatis* used 12.5 μl of 2× TB Green Premix Ex Taq GC, 0.05 μM of each primer, 9.5 μl of water, and 2 μl of genomic DNA (20 ng). Conditions were 95°C for 30 seconds (s), followed by 35 cycles of 95°C for 5 s and 60°C for 30 s. Fluorescence was measured during elongation, and melting curve analysis was automatic. Data were analysed with default software settings. Copy numbers of *tuf* were determined from standard curves. The relative abundance of each bacterium was calculated using the 2−ΔCt method, where ΔCt = ‘average Ct of target’ − ‘average Ct of *tuf*’.

**Table 1:** Primers used for quantitative polymerase chain reaction

Target	Primer sequences	Reference
*Fusobacterium nucleatum*	F: CAACCATTACTTTAACTCTACCATGTTCA R: GTTGACTTTACAGAAGGAGATTATGTAAAAATC	^10^
*Parvimonas micra*	F: AACGACGATTAATACCGCATGAGACC R: CTTCCTCCTATGATACCGTCATTA	^26^
*Peptostreptococcus stomatis*	F: CGGCAGCAGGATACATAGC R: TGGACAAGGAGTGGTAGGTT	^27^

### Statistical analysis

Analyses were performed with R, EZR, and GraphPad Prism. Categorical variables were compared with χ^2^ or Fisher’s exact tests. Continuous variables were analysed with the Student’s *t*-test, Mann–Whitney *U* test, Wilcoxon signed-rank test, or Kruskal–Wallis test, followed by Steel–Dwass *post hoc* testing. LMMs were fitted with time point as a fixed effect and patient ID as a random effect to account for repeated measurements. Overall time point effects were assessed using likelihood ratio tests comparing full models (including time point) with corresponding null models (excluding time point), fitted using maximum likelihood (REML = FALSE). Pairwise comparisons were performed using estimated marginal means, with *P* values adjusted using the Benjamini–Hochberg procedure when applicable. Adjusted *P* values < 0.050 were considered statistically significant. This LMM framework was applied to alpha diversity indices, taxonomic relative abundances, and qPCR data.

## Results

### Patients’ characteristics

A total of 83 faecal samples were collected from 28 patients with pathologically confirmed CRC undergoing curative laparoscopic resection, and from 16 age- and sex-matched healthy controls. A total of 27 preoperative samples, 11 at 1 week, 17 at 1 month, and 12 at 6 months, were analysed. There were no significant differences in age or sex between patients and controls (*Table 2*).

**Table 2:** Demographic and clinical characteristics

Baseline characteristics	CRC (*n* = 28)	Control (*n* = 16)	P*
Age (years), mean(s.d.)	66.8(12.3)	63.4(5.0)	0.290
**Sex**			0.760
Male	12 (43%)	6 (37%)	
Female	16 (57%)	10 (63%)	
**Timing**			
Preoperative	27 (96%)		
1 week	11 (39%)		
1 month	17 (61%)		
6 months	12 (43%)		
**Tumour location**			
Ascending	9 (32%)		
Transverse	2 (7%)		
Descending	1 (4%)		
Sigmoid	6 (21%)		
Rectum	10 (36%)		
**Resection**			
Right	11 (39%)		
Left	17 (61%)		
**T category**			
T1	9 (32%)		
T2	3 (11%)		
T3	13 (46%)		
T4	3 (11%)		
**N category**			
N0	17 (61%)		
N1	6 (21%)		
N2	3 (11%)		
N3	2 (7%)		
**M category**			
M0	28 (100%)		
M1	0 (0%)		
**Tumour stage**			
I	11 (39%)		
II	6 (21%)		
III	11 (39%)		
IV	0 (0%)		
**Recurrence**			
Peritoneum	1 (4%)		
Liver	1 (4%)		
Lung	1 (4%)		
CEA (ng/ml), median (i.q.r.)	3.2 (2.08–4.8)		
CA19-9 (U/ml), median (i.q.r.)	10 (5.75–26.25)		

Values are *n* (%) unless otherwise stated. CRC, colorectal cancer; s.d., standard deviation; NA, not applicable; CEA, carcinoembryonic antigen; CA19-9, carbohydrate antigen 19-9; i.q.r. interquartile range. *Continuous variables were analysed using Student's *t*-test, and categorical variables using the chi-square test.

### Longitudinal changes in gut microbiota diversity

**Fig. 1:**
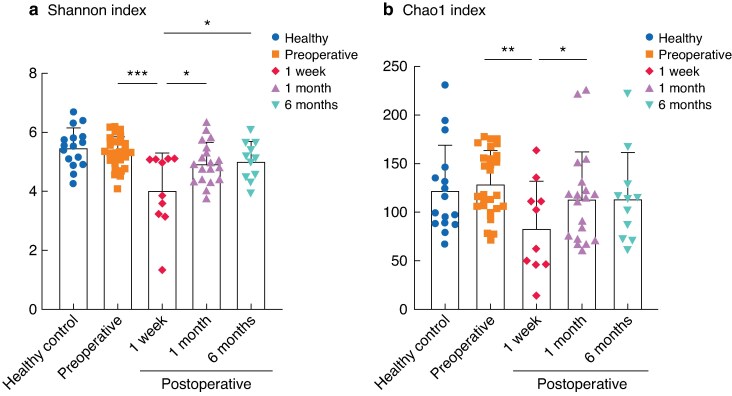
Alpha diversity across groups **a** Shannon diversity index, **b** Chao1 richness index. Each dot represents an individual sample, and bars indicate group means with whiskers denoting standard deviations. Healthy controls represent a single time point reference group. Healthy controls were not included in statistical comparisons and are shown for reference only. *P < 0.050, **P < 0.010, ***P < 0.001. Both indices were compared using linear mixed-effects models with timepoint as a fixed effect and patient ID as a random effect; pairwise comparisons were performed using estimated marginal means with Benjamini–Hochberg adjustment.

Alpha diversity was assessed in the healthy control, preoperative, 1 week, 1 month, and 6 month groups using Shannon and Chao1 indices. The 1 week group showed a significantly lower Shannon index (*P* < 0.050, *Fig. 1a*) and the Chao1 index showed a similar trend (*Fig. 1b*). By 1 month after surgery, both indices had returned to baseline levels. The healthy controls, who were sampled only once, exhibited alpha diversity values comparable to the preoperative samples and were included as an independent reference group.

**Fig. 2:**
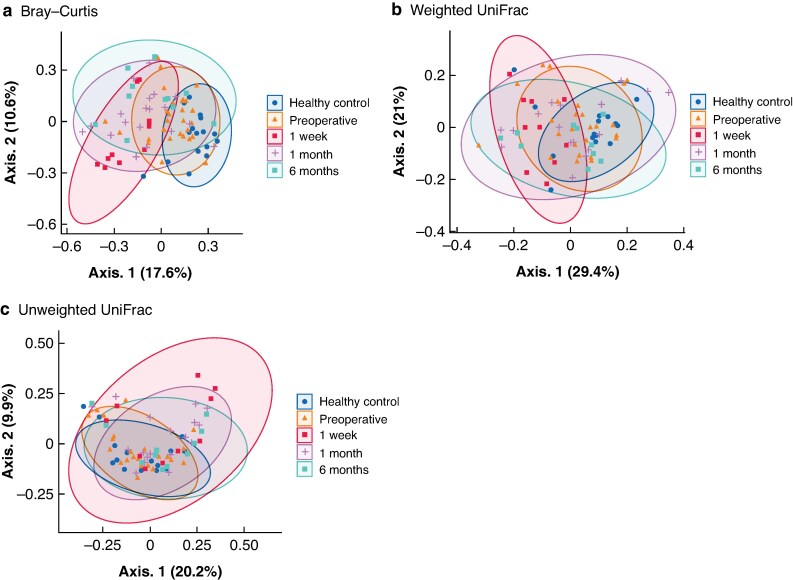
PCoA based on genus-level beta diversity distances among study groups **a** Weighted UniFrac distance, **b** Unweighted UniFrac distance, **c** Group-level differences, all assessed using PERMANOVA followed by pairwise comparisons. Each dot represents an individual sample. PCoA was performed using Bray–Curtis distance. PCoA, principal coordinate analysis.

Genus-level beta diversity was assessed using Bray–Curtis, weighted UniFrac, and unweighted UniFrac distance metrics to characterize postoperative alterations in gut microbial communities. Detailed results of the pairwise PERMANOVA are provided in *Table S1*. Bray–Curtis analysis (*Fig. 2a*) demonstrated that both the healthy control and 1 week groups differed significantly from all other groups. In addition, significant differences were observed between the preoperative and 1 month/6 month groups, whereas no significant differences were detected among the postoperative groups (1 week, 1 month, and 6 months). These findings indicate that the relative abundances of bacterial taxa changed markedly immediately after surgery and remained quantitatively distinct from the preoperative state at later time points, despite relative stability within the postoperative period. Weighted UniFrac analysis (*Fig. 2b*) revealed a significant difference between the preoperative and 1 week groups. However, the distances did not differ significantly between the 1 week and 1 month/6 month groups. This suggests that the composition of dominant bacterial lineages did not undergo further large-scale shifts beyond the early postoperative phase. Unweighted UniFrac analysis (*Fig. 2c*) showed significant differences between the preoperative and 1 week/1 month groups. In contrast, no significant difference was detected between the preoperative and 6 month groups, and the postoperative groups did not differ significantly from each other. This pattern is consistent with a gradual recovery of community membership towards the preoperative state, as assessed by presence–absence-based metrics.

Taken together, these results indicate an acute postoperative perturbation of the gut microbiota, followed by stabilization of overall bacterial lineage composition over time. Moreover, differences in relative abundance profiles persisted at later postoperative time points, indicating ongoing community reorganization rather than a simple return to the preoperative state.

### Changes in the relative abundance of gut microbiota at different taxonomic levels

**Fig. 3:**
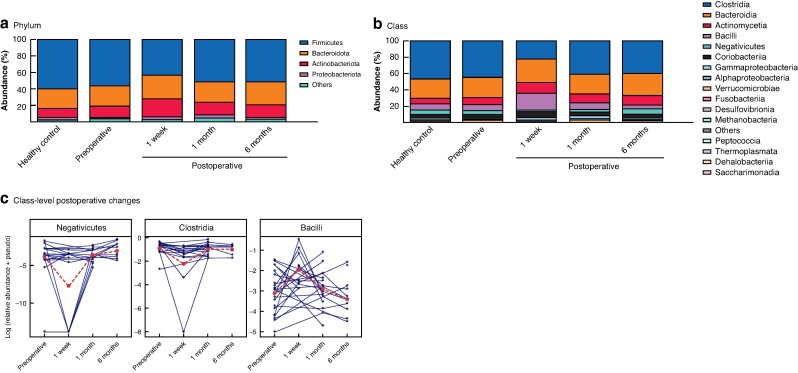
Bacterial composition of each group at the phylum and class levels, and class level postoperative changes **a** Phylum, **b** Class levels. Each bar represents the percentage composition of bacteria. The top three taxa exhibiting the largest time-associated changes from the preoperative to 6 month postoperative time points, as estimated using LMMs, are shown at the class level. **c** Taxa are presented based on the magnitude of estimated temporal change for descriptive purposes, and no formal differential abundance testing was performed.

The overall bacterial compositions at the phylum and class levels are shown in *Fig. 3a,b*. At the phylum level, postoperative changes were most apparent for Firmicutes, with Actinobacteriota showing comparatively smaller variations (*Fig. 3a*). At the class level, apparent postoperative alterations were observed for Clostridia and Bacilli, which showed noticeable changes in relative abundance at 1 week, followed by a gradual return toward preoperative levels at 1 and 6 months (*Fig. 3b*). To characterize class-level dynamics further, class-level taxa were ranked according to the magnitude of postoperative change, and the top three classes were examined. All three belonged to the phylum Firmicutes; notably, Negativicutes and Clostridia exhibited a decrease at 1 week followed by a gradual recovery towards later postoperative time points, whereas Bacilli displayed a distinct pattern, with a transient increase at 1 week and subsequent recovery (*Fig. 3c*).

Within Bacilli, genera showing the largest magnitude of postoperative change included *Enterococcus* and *Lactobacillus*, as summarized in *Table S2*.

### Identification and targeted relative quantification of CRC-associated bacteria

**Fig. 4:**
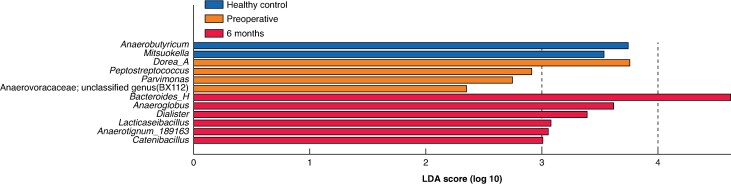
Differentially abundant bacterial genera among the healthy control, preoperative, and 6 month groups identified by LEfSe LEfSe was used to calculate scores based on relative abundances. The analysis was performed at the genus level. These results are presented as exploratory cross-sectional findings. Bacterial genera with LDA scores > 2.0 and nominal *P* < 0.05 based on the Kruskal–Wallis test followed by pairwise Wilcoxon tests are shown. LDA, linear discriminant analysis; LEfSe, linear discriminant analysis effect size.

**Fig. 5:**
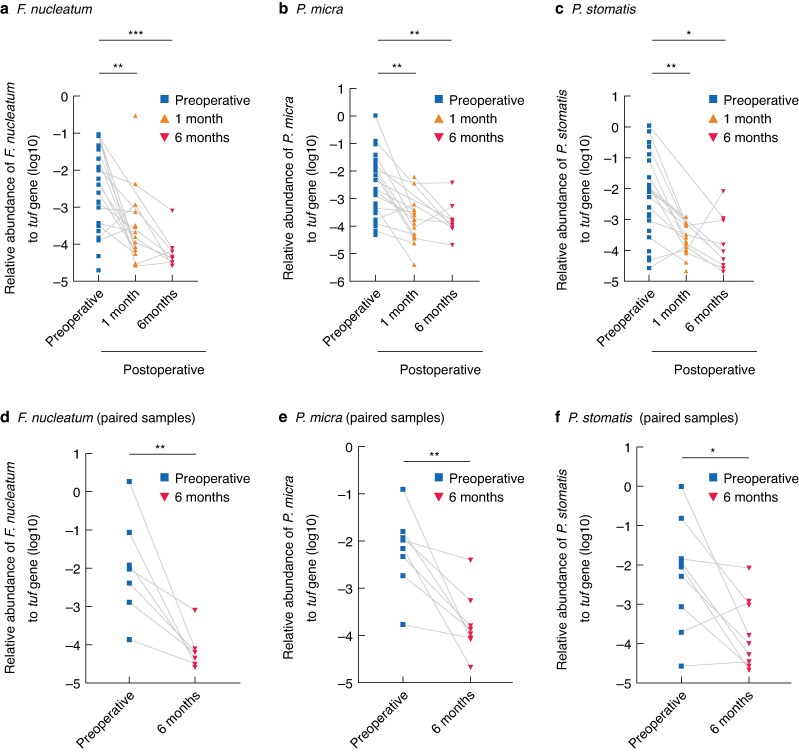
Relative abundances of *F. nucleatum*, *Parvimonas micra*, and *Peptostreptococcus stomatis* relative to the *tuf* gene, measured by qPCR **a–c** Relative abundances of *F. nucleatum*, *P. micra*, and *P. stomatis* in all samples from the preoperative, 1 month, and 6 month groups, **d–f** relative abundances of *F. nucleatum*, *P. micra*, and *P. stomatis* in paired preoperative and 6 months samples. Each dot represents a sample. Horizontal lines indicate medians. Samples from the same individuals are shown as connected points. Statistical analysis was performed using linear mixed-effects models with likelihood ratio tests and pairwise comparisons adjusted using **a–c** the Benjamini–Hochberg procedure and **d–f** Wilcoxon signed-rank test (*P < 0.050, **P < 0.010, ***P < 0.001). qPCR, quantitative polymerase chain reaction.

To complement the longitudinal analyses, LEfSe analysis was performed to identify genera differentially enriched across perioperative groups. LEfSe analysis (LDA score > 2.0, *P* < 0.050) identified 12 genera with significant intergroup differences. Among these, *Peptostreptococcus* and *Parvimonas* were enriched in the preoperative group (*Fig. 4*). Given that specific species within these genera—namely *P. stomatis* and *P. micra*—have been consistently reported as CRC-associated bacteria in previous studies^10^, targeted qPCR analysis was performed to assess quantitatively their perioperative dynamics. In addition, *F. nucleatum* was included, a well established CRC-associated bacterium, for parallel qPCR analysis based on strong previous evidence^12,27^. The reference *tuf* gene did not differ among groups (*P* = 0.290; *Fig. S2*), indicating comparable total bacterial load across samples and confirming its suitability as an internal control. Relative abundances of *F. nucleatum*, *P. micra*, and *P. stomatis* decreased significantly at 1 and 6 months compared with levels in the preoperative group (*P* < 0.050 for all; *Fig. 5a–c*). In paired analyses, all three bacteria showed significant reductions between samples collected in the preoperative group and those in the 6 month group (*P* < 0.050 for all; *Fig. 5d–f*).

Among the three patients who developed recurrence (*Table 2*), preoperative levels of *P. micra* and *P. stomatis* were significantly higher than those in patients without recurrence, whereas *F. nucleatum* showed no difference (*P* = 0.990; *Fig. S3a–c*). At 1 month, these differences were no longer observed (*P. micra*: *P* = 0.940; *P. stomatis*: *P* = 0.320; *Fig. S3d,e*).

## Discussion

This prospective study demonstrated that curative CRC resection induced an acute postoperative disruption of the gut microbiota, followed by recovery of overall alpha diversity. In contrast, beta diversity analyses revealed stabilization into an alternative microbial state rather than a simple return to the preoperative structure. Importantly, CRC-associated bacteria, including *F. nucleatum*, *P. micra*, *and P. stomatis*, remained suppressed for at least 6 months after resection. These findings highlight the close dependence of CRC-associated bacteria on the tumour microenvironment and demonstrated that ecological alterations in the gut microbiota persist beyond short-term perioperative perturbations. To the authors’ knowledge, this is the first longitudinal study to characterize both short- and long-term alterations in the gut microbiota, focusing on these taxa, during the perioperative period, providing novel insights into how tumour-associated microbes behave following curative CRC resection.

Shannon and Chao1 indices showed that colorectal resection induced a marked and rapid perturbation of the gut microbiota at 1 week, reflecting an acute loss of microbial richness and evenness, followed by recovery to near-preoperative levels by 1 month (*Fig. 1*). At the same time, visualization of higher-level taxonomic composition indicated partial recovery of several major bacterial groups towards preoperative relative abundances, giving the overall impression of microbial recovery following surgery (*Fig. 3a,b*). Closer inspection at the class level, however, revealed non-uniform recovery. Although all these classes belong to the phylum Firmicutes, Negativicutes and Clostridia exhibited a marked decrease at 1 week followed by gradual recovery towards later time points, whereas Bacilli showed an opposite trajectory (*Fig. 3c*). Interestingly, genus-level analyses within Bacilli indicated that this increase was driven primarily by *Enterococcus* and *Lactobacillus* (*Table S2*). These patterns likely reflect the known effects of β-lactam antibiotics and mechanical bowel preparation. These interventions transiently reduce microbial diversity and suppress Clostridia. At the same time, they enrich Bacilli, including β-lactam-resistant genera such as *Enterococcus*, for several weeks.^13,28^. The transient increase in *Lactobacillus* at 1 week may reflect its facultative anaerobic nature, allowing it to expand relative to other taxa under oxygen-exposed perioperative conditions^29^. The subsequent decline towards 6 months suggests that these conditions are temporary and that the intestinal environment gradually returns to a more stable anaerobic state.

In beta diversity analyses, unweighted UniFrac revealed an acute postoperative perturbation of the gut microbiota, followed by restoration of overall community membership. In contrast, Bray–Curtis and weighted UniFrac metrics, which profile relative abundances, remained altered at later postoperative time points, indicating community reorganization rather than a return to the preoperative state (*Fig. 2*). One possible explanation is that numerous small residual deviations in genus-level relative abundances persisted over time, and their aggregate effect was reflected in abundance-based beta diversity metrics.

In line with this notion of community reorganization, CRC-associated bacteria showed a distinct postoperative pattern. LEfSe and qPCR analyses confirmed that *F. nucleatum*, *P. micra*, and *P. stomatis* declined significantly by 1 month and remained suppressed at 6 months after surgery (*Figs 4*, *5*). This pattern is consistent with findings from large Japanese cohorts using shotgun metagenomics^12,27^. These oral-origin bacteria are consistently enriched in patients with CRC across diverse populations, suggesting a strong association with tumour presence^30,31^. Their behaviour is consistent with the driver–passenger model: early ‘driver’ bacteria initiate tumorigenesis through DNA damage or immune activation, whereas ‘passenger’ bacteria preferentially colonize and adapt to the tumour microenvironment^32,33^. *F. nucleatum*, for example, adheres to malignant epithelial cells via FadA and Fap2, promoting tumour progression^3,9,34,35^. In line with this, *F. nucleatum*, *P. micra*, and *P. stomatis* increase in abundance in more advanced CRC stages^27^. Evidence also indicates that these organisms adapt selectively to the tumour niche^36^. Certain *P. micra* strains preferentially colonize tumour tissue^37^, and *F. nucleatum* strains isolated from saliva and tumours are genetically identical at the strain level^38^. Moreover, tumour-derived *P. micra* strains exhibit upregulated pathways involved in inflammation and adhesion, suggesting functional adaptation to the tumour microenvironment^38^. Taken together, the sustained postoperative suppression of these bacteria after curative CRC resection reinforces the concept that they cannot persist without a tumour-supportive niche, consistent with their classification as passenger bacteria in CRC.

Preoperative levels of *P. micra* and *P. stomatis* were higher in patients who later developed recurrence compared with non-recurrent patients (*Fig. S3b,c*). However, these differences were no longer observed at 1 month (*Fig. S3d,e*). This suggests that, although elevated preoperative levels may be present in high-risk patients, their prognostic value remains uncertain once the tumour is removed. Previous studies have linked intratumoral *P. micra* to microsatellite instability and *BRAF* mutations, both markers of poor prognosis^39^, and high levels of *F. nucleatum* in tumours after neoadjuvant chemoradiotherapy to recurrence and poor outcomes^8^. These observations support the notion that such bacteria may influence progression and recurrence risk. Whether perioperative stool-based measurements can serve as reliable biomarkers therefore depends on whether persistent elevation or insufficient reduction in levels of *F. nucleatum*, *P. micra*, or *P. stomatis* predicts recurrence. The authors’ findings remain exploratory, as only three patients in this cohort developed recurrence. Future large-scale, multicentre longitudinal studies will be required to validate whether specific bacterial patterns predict outcomes after curative CRC resection. However, with further validation, stool-based monitoring of these bacteria may serve as non-invasive biomarkers for perioperative risk stratification, surveillance of microbiota recovery, and early detection of recurrence.

This study has several limitations. First, faecal samples were not consistently obtained from the same individuals at all time points. Limited patient compliance and clinical scheduling factors, including initiation of adjuvant chemotherapy or detection of recurrence, reduced the feasibility of uniform longitudinal sampling. This resulted in unbalanced longitudinal data and may have introduced interpersonal variability, complicating interpretation of gut microbiota alterations over time. To address this issue, longitudinal analyses were performed using statistical approaches that explicitly account for repeated measures and missing observations, including LMMs with subject-level random effects. Whereas these methods mitigate bias arising from non-independence and incomplete sampling, they cannot fully substitute for complete patient-matched longitudinal data, and subtle within-subject temporal patterns may therefore have been missed. Such efforts, potentially combined with advanced analytical frameworks for complex longitudinal data, will be essential for accurately characterizing perioperative microbial alterations and for evaluating their potential clinical relevance in CRC. Second, only a small number of patients developed recurrence during follow-up, which limited statistical power to assess associations between microbial alterations and clinical outcomes. Third, perioperative factors such as mechanical bowel preparation, antibiotic exposure, and dietary variability were not fully controlled and may have influenced microbiota composition. Future studies should adopt fully patient-matched longitudinal sampling protocols, recruit larger cohorts, and ideally incorporate multicentre validation.

## Supplementary Material

zrag037_Supplementary_Data

## Data Availability

Data supporting the findings of this study are available from the corresponding author upon reasonable request.
