# Supplementary material for: Short- and long-term dynamics of gut microbiota, highlighting Fusobacterium nucleatum, Parvimonas micra, and Peptostreptococcus stomatis after colorectal cancer resection: prospective cohort study
Source: BJS Open. 2026 May 21;10(3):zrag037. doi: 10.1093/bjsopen/zrag037 (PMC13195633; doi:10.1093/bjsopen/zrag037)
Supplement: zrag037_Supplementary_Data [file zrag037_supplementary_data.docx]

**Short- and Long-Term Dynamics of Gut Microbiota, Highlighting Fusobacterium nucleatum, Parvimonas micra and Peptostreptococcus stomatis After Colorectal Cancer Resection**

TOMOSUKE MUKOYAMA^1^, KIMIHIRO YAMASHITA^1,2^, MASAFUMI SAITO^3^, MITSUGU FUJITA^4^, SEIICHI OMURA^5^, TAKUO EMOTO^6^, TOMOYA YAMASHITA^6,7^, MASAYUKI ANDO^1^, KYOSUKE AGAWA^1^, KOTA YAMADA^1^, AKIHIRO WATANABE^1^, TOMOKI ABE^1^, TAKAO TSUNEKI^1^, YUKARI ADACHI^1^, RYUICHIRO SAWADA^1^, YASUFUMI KOTERAZAWA^1^, HITOSHI HARADA^1^, NAOKI URAKAWA^1^, HIRONOBU GOTO^1^, HIROSHI HASEGAWA^1^, SHINGO KANAJI^1^, TAKERU MATSUDA^1^, TARO OSHIKIRI^1^, YOSHIHIRO KAKEJI^1^

1. Division of Gastrointestinal Surgery, Department of Surgery, Graduate School of Medicine, Kobe University, Kobe, Hyogo, Japan
2. Department of Biophysics, Kobe University Graduate School of Health Sciences, Kobe, Japan.

3.　Department of Immunology and Microbiology, National Defense Medical College, Tokorozawa, Japan

4.　Center for Medical Education and Clinical Training, Kindai University Faculty of Medicine, Osaka-Sayama, Osaka, Japan

5.　Department of Microbiology, Faculty of Medicine, Kindai University, Osaka-Sayama City, Osaka, Japan

6.　Division of Cardiovascular Medicine, Department of Internal Medicine, Kobe University Graduate School of Medicine, Kobe, Japan

7.　Division of Advanced Medical Science, Technology and Innovation, Kobe University Graduate School of Science, Kobe, Japan.

**Corresponding author.**

Kimihiro Yamashita

7-5-2, Kusunoki-cho, Chuo-ku, Kobe, Hyogo, Japan

**Supplementary Materials - Index**

| **Supplementary Figures and Tables** |  |  |
| --- | --- | --- |
| Supplementary Figure 1 |  | *pag. 2* |
| Supplementary Figure 2  Supplementary Figure 3 |  | *pag. 3*  *pag. 4* |
| Supplementary Table 1  Supplementary Table 2 |  | *pag.5*  *pag.6* |
|  |  |  |

**Supplementary Figures**

**Supplementary Figure 1**

**
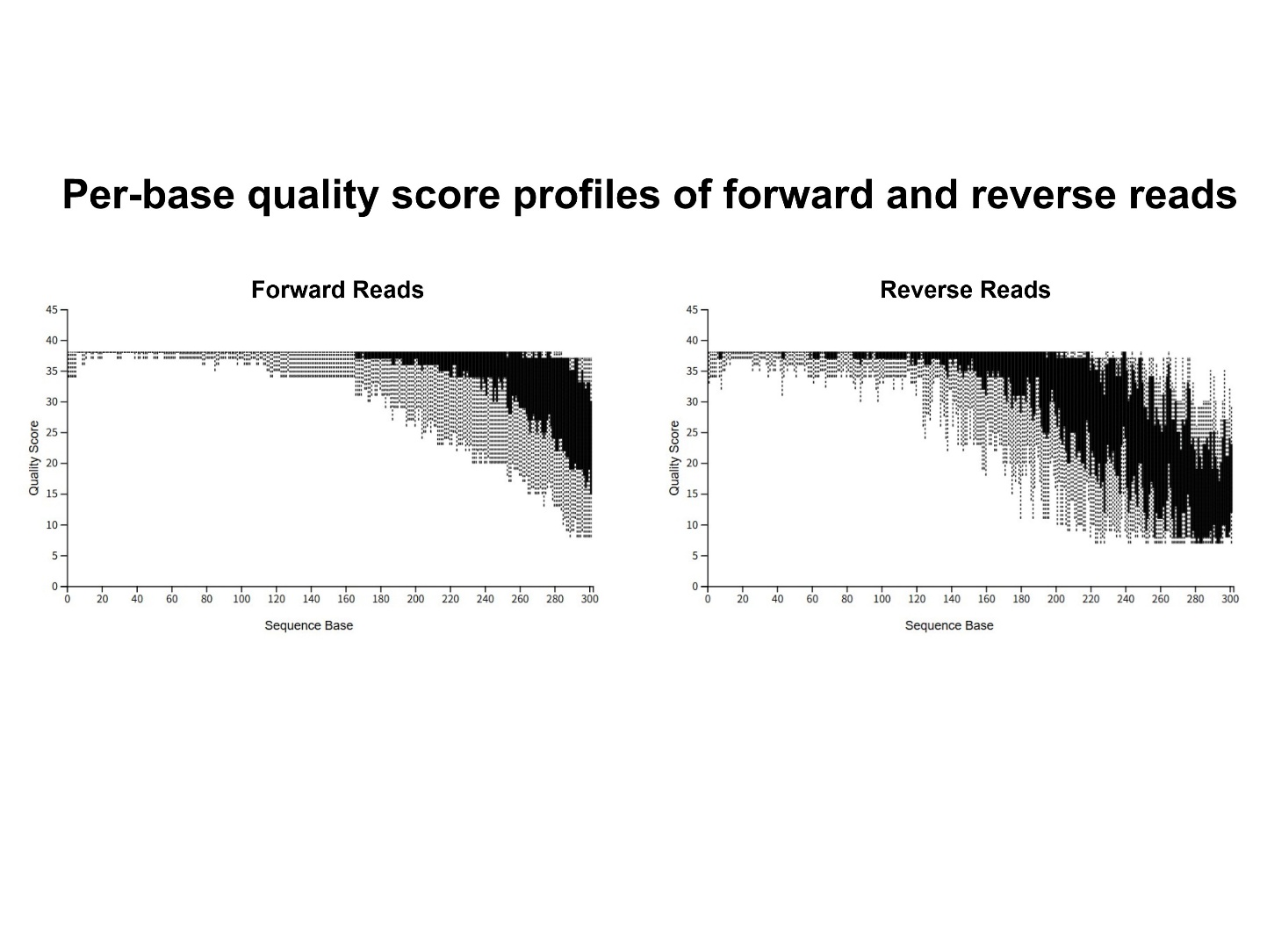
**

**Supplementary Figure 2**

**
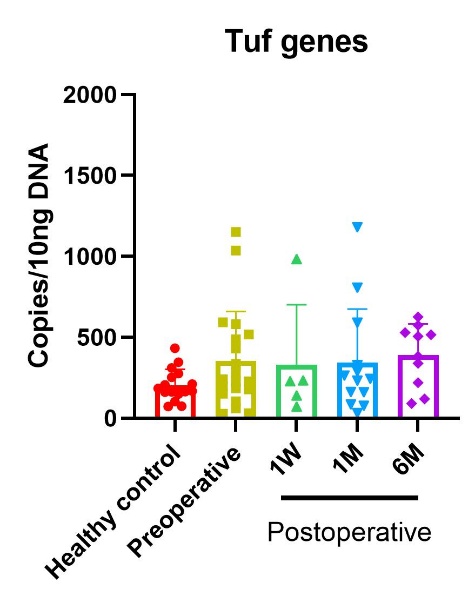
**

**Supplementary Figure 3**

**
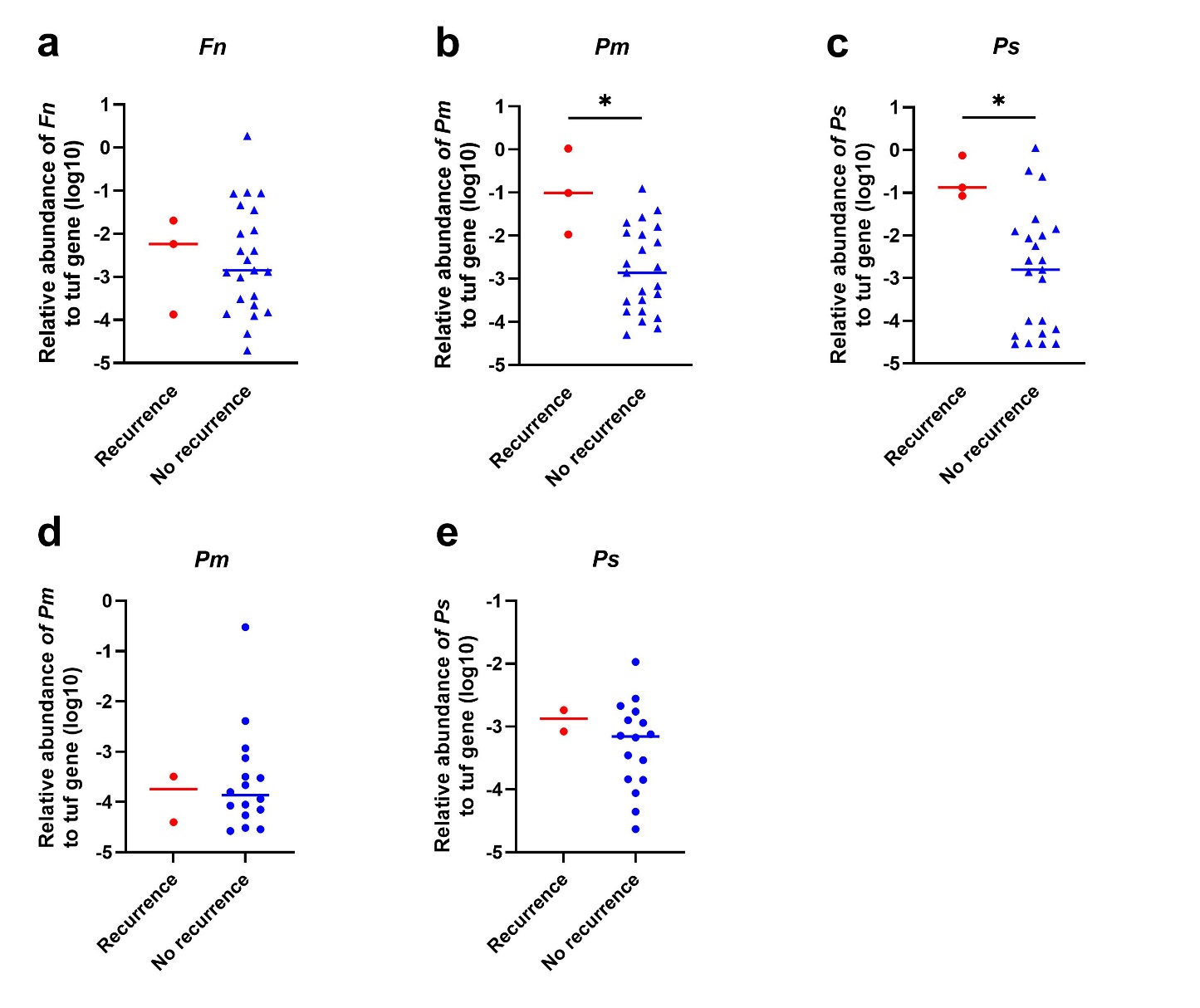
**

| **Supplementary Table 1.**  **Pairwise PERMANOVA results for β-diversity analyses** | | | |
| --- | --- | --- | --- |
| **Groups** | **R²** | ***p* value** | ***q* value** |
| **Bray-Curtis** |  |  |  |
| Healthy control vs Preoperative | 0.050 | 0.004 | 0.010 |
| Healthy control vs 1W | 0.200 | <0.001 | <0.010 |
| Healthy control vs 1M | 0.120 | <0.001 | <0.010 |
| Healthy control vs 6M | 0.150 | <0.001 | <0.010 |
| 1W vs Preoperative | 0.120 | <0.001 | <0.010 |
| 1W vs 1M | 0.060 | 0.028 | 0.030 |
| 1W vs 6M | 0.130 | 0.005 | 0.010 |
| 1M vs Preoperative | 0.050 | 0.006 | 0.010 |
| 1M vs 6M | 0.030 | 0.462 | 0.460 |
| 6M vs Preoperative | 0.050 | 0.029 | 0.030 |

**Weighte UniFrac**

| Healthy control vs Preoperative | 0.030 | 0.290 | 0.420 |
| --- | --- | --- | --- |
| Healthy control vs 1W | 0.160 | 0.003 | 0.020 |
| Healthy control vs 1M | 0.040 | 0.140 | 0.230 |
| Healthy control vs 6M | 0.070 | 0.095 | 0.190 |
| 1W vs Preoperative | 0.110 | 0.002 | 0.020 |
| 1W vs 1M | 0.070 | 0.071 | 0.180 |
| 1W vs 6M | 0.120 | 0.032 | 0.110 |
| 1M vs Preoperative | 0.020 | 0.503 | 0.560 |
| 1M vs 6M | 0.020 | 0.655 | 0.660 |
| 6M vs Preoperative | 0.030 | 0.398 | 0.500 |

**Unweighted UniFrac**

| Healthy control vs Preoperative | 0.030 | 0.127 | 0.160 |  |
| --- | --- | --- | --- | --- |
| Healthy control vs 1W | 0.110 | 0.003 | 0.020 |  |
| Healthy control vs 1M | 0.070 | 0.006 | 0.020 |  |
| Healthy control vs 6M | 0.070 | 0.059 | 0.120 |  |
| 1W vs Preoperative | 0.080 | 0.003 | 0.020 |  |
| 1W vs 1M | 0.050 | 0.092 | 0.130 | |
| 1W vs 6M | 0.060 | 0.292 | 0.320 |  |
| 1M vs Preoperative | 0.040 | 0.013 | 0.030 |  |
| 1M vs 6M | 0.030 | 0.680 | 0.680 |  |
| 6M vs Preoperative | 0.040 | 0.069 | 0.120 |  |

Abbreviations: 1W, 1 week; 1M, 1 month; 6M, 6 months

| **Supplementary Table 2. Top three genera within the class *Bacilli* exhibiting large postoperative shifts** | | | | | | |  |
| --- | --- | --- | --- | --- | --- | --- | --- |
| **Genus** | **Pre** | **1W** | **6M** | **\|Δ Pre–1W\|** | **\|Δ1W–6M\|** | **Bidirectional change score** | |
| Enterococcus_H | -12.840 | -6.223 | -13.237 | 6.617 | 7.014 | 6.617 | |
| Enterococcus_B | -12.603 | -10.216 | -12.259 | 2.387 | 2.043 | 2.043 | |
| Lactobacillus | -10.771 | -6.951 | -8.815 | 1.890 | 2.740 | 1.890 | |
| Values are shown as log-transformed relative abundances (log [relative abundance + 1×10⁻⁶]). Bidirectional change score was defined as min (\|ΔPre–1W\|, \|Δ1W–6M\|). Abbreviations: Pre, Preoperative; 1W, 1 week; 1M, 1 month; 6M, 6 months | | | | | | | |
